# Enhancing drought resistance in *Pogostemon cablin* (Blanco) Benth. through overexpression of ACC deaminase gene using thin cell layer regeneration system

**DOI:** 10.3389/fpls.2023.1238838

**Published:** 2023-08-11

**Authors:** Zafar I. Warsi, Kahkashan Khatoon, Pooja Singh, Laiq Ur Rahman

**Affiliations:** Central Institute of Medicinal and Aromatic Plants, Council of Scientific and Industrial Research (CSIR), Lucknow, India

**Keywords:** *Pogostemon cablin*, direct regeneration, genetic transformation, tTCLs, ACC deaminase, molecular docking, drought resistance

## Abstract

*Pogostemon cablin* cultivation faces massive constraints because of its susceptability to drought stress that reduces patchouli propagation and oil yield. The present study has achieved an efficient and rapid direct regeneration system for the transgenic production of *P. cablin* using Agrobacterium-mediated genetic transformation. To establish an efficient regeneration protocol for fast *in-vitro* multiplication of patchouli plants, leaf, petiole, and transverse thin cell layer (tTCL) explants were used and inoculated on an MS medium supplemented with different combinations of phytohormones. A comparative study showed a maximum regeneration frequency of 93.30 ± 0.56% per explant was obtained from leaf segments on optimal MS medium fortified with 0.2mg/L BAP and 0.1mg/L NAA. Leaf and petiole explants took 25-35 days to regenerate while tTCL section showed regeneration in just 15-20 days on the same medium. Subsequently, productive genetic transformation protocol OD_600_ 0.6, AS 200µM, 30mg/L kanamycin, and infection time 5 min. was standardized and best-suited explants were infected at optimum conditions from the *Agrobacterium tumefaciens* (LBA 4404) strain harboring *ACC deaminase* to generate transgenic *P. cablin* Benth. (CIM-Samarth) plants. The investigation suggested that the optimized protocol provides a maximum transformation frequency of 42 ± 1.9% in 15-20 days from tTCL. The transgenic plants were shifted to the greenhouse with a 52.0 ± 0.8% survival frequency. A molecular docking study confirmed significant binding affinity of ligand ACC with *ACC deaminase* at the catalytic site, and ligand interactions showed four H-bonds at the binding pocket with amino acids Cys-196, Val-198, Thr-199, and Gly-200 that validate gene relative expression in transgenic plants. Among all transgenic acclimatized greenhouse-grown patchouli plants, line PT4 showed improved drought resistance under severe water stress as its RWC was 71.7 ± 2.3% to 75.7 ± 2.1% which is greater than the RWC of the control plant, 58.30 ± 0.21%. Analysis of the other physiological indicators, H_2_O_2_, chlorophyll content, and ROS result support drought resistance ability. Our study concluded that the first report on *P. cablin*, tTCL direct regeneration, and standardized transformation protocol created a new opportunity for genetic manipulation to achieve drought-resistant patchouli plants for cultivation in all seasons at the commercial level.

## 1 Introduction

The plant patchouli *(Pogostemon cablin.*, family Lamiaceae); is an essential aromatic herb, native to tropical regions of Asia (Wang et al., 2019). Patchouli oil (PO) is commercially utilized in the perfumery, cosmetic, and aromatherapy industries worldwide. Besides being used in cosmetics, it is used as an antidepressant, antiphlogistic, antiseptic, aphrodisiac, astringent, diuretic, febrifuge, and fungicide (Miyazawa et al., 2000; Bunrathep et al., 2006). The major component of patchouli essential oil are patchoulene, caryophyllene, pogostol, guaiene, patchoulol, and α-patchoulene (Zhou et al., 2011; Liu et al., 2015). Patchoulol and α- patchoulene are responsible for maintaining the quality of PO (Ramya et al., 2013; Pandey et al., 2021). The potential application of PO in pharmacology and agriculture has led to an enormous global demand. The estimated global patchouli oil production is 800 tonnes/year, about 60% of which is produced by Indonesia (Tahir et al., 2019). Patchouli cultivation in India is approximately 600 ha, producing 20 tonnes of oil/annum (Choudhri et al., 2023).

Global climatic changes impose a wide range of stresses over crop cultivation and production (Chakraborty et al., 2014; Roychowdhury, 2014; Roychowdhury et al., 2020). The cultivation of shade-loving patchouli faces significant constraints due to its susceptibility to various biotic stresses (pests, fungi, viruses, and root-knot nematodes) and abiotic stresses (Singh et al., 2009; Bhau et al., 2016; Suhesti et al., 2022). In Southeast Asia, patchouli cultivation dominantly occurs in dry land therefore, *P. cablin* faces drought stress which limits its rapid growth and productivity (Suhesti et al., 2022). These stresses induce excess ethylene synthesis, resulting in senescence, abscission, and leaf yellowing ultimately reducing its essential oil yield (Glick, 2014). Until now, among all the major varieties of patchouli, none of them are showing improved drought tolerance capability. Consequently, preference has been given to developing varieties resistant/tolerant to drought stress with high biomass and better PO quality (Suhesti et al., 2022). Moreover, various conventional propagation methods cannot be practiced for genetic modification of *P. cablin-*resistant varieties because of the low transformation efficiency. Therefore, biotechnological applications could be successful in performing *Agrobacterium tumefaciens-* mediated genetic transformation with specific genes (Roychowdhury and Tah, 2013; Roychowdhury et al., 2023). The genetic manipulation provides a new dimension to develop superior patchouli drought resistant lines to meet the industrial demand of PO by cultivating in all seasons (Paul et al., 2012).

Recently, biotechnological techniques have been developed for microbial-plant interactions to utilize beneficial micro-biome characteristics for improving plant quantity and quality (Moon and Ali, 2022; Li et al., 2023a; Li et al., 2023b). One such technique is using *ACC deaminase*-PGPR for regenerative agriculture, which has shown to be highly advantageous (Glick, 2014; Han et al., 2021). During stress, plants produce excess ethylene called ‘stress ethylene’, which can impair primary metabolism (Singh et al., 2021). Drought stress compromises various physiological and metabolic pathways, resulting in stunted plant growth, interrupted photosynthesis, and abnormal metabolism leading to plant death (Hasanuzzaman et al., 2015; Anumalla et al., 2016; Zhang et al., 2018). As per the literature study, PGPRs that produce *ACC deaminase* have been found to reduce stress ethylene phytohormone levels under biotic and abiotic stresses (Penrose & Glick, 2003) by catalyzing ACC into α-ketobutyrate and ammonia (Li et al., 2019). It is reported by Glick (2014) that the transformation of the bacterial *ACC deaminase* gene supports reducing ethylene concentration and improving root permeability along with nitrate availability. Therefore, *Agrobacterium*-mediated genetic engineering is crucial for decreasing ethylene accumulation without affecting plant growth and development.

To genetically improve *P. cablin*, an efficient, fast, and highly reproducible regeneration system has been acquired in the present research to transform the *ACC deaminase* gene for large-scale cultivation in every season. Van (1973) developed the thin cell layer (TCL) technique, a low-cost, fast multiplication, and highly reproducible *in-vitro* propagation method for large-scale production of genetically stable plants. Either longitudinal (lTCL) or transverse (tTCL) section of ~0.1-5mm thin explants can be used for the propagation and preservation of various significant plants, including endangered ones (Rout et al., 2006; Singh et al., 2012; Zhao et al., 2007). Although regeneration and transformation protocols have been reported in *P. cablin* (Paul et al., 2010; Paul et al., 2012; Sugimura et al., 2005) from callus and direct regeneration from leaf disc, the transformation parameters were not well-concluded to provide better variety.

By keeping the detailed literature in mind, the present study demonstrates the establishment of an efficient direct regeneration system from leaf, petiole, and internodal transverse thin cell layer (tTCL) explants of patchouli to avoid somaclonal variation for large-scale multiplication in every season. The consequence of fast regeneration provides a smooth platform to address *Agrobacterium tumefaciens-*mediated genetic transformation with a construct harboring the *ACC deaminase* gene to analyse the tolerance level of acclimatized greenhouse-grown transgenic *P. cablin* plants against drought stress.

## 2 Materials and methods

### 2.1 Establishment of *in vitro* and direct regeneration protocols

**Figure 1:**
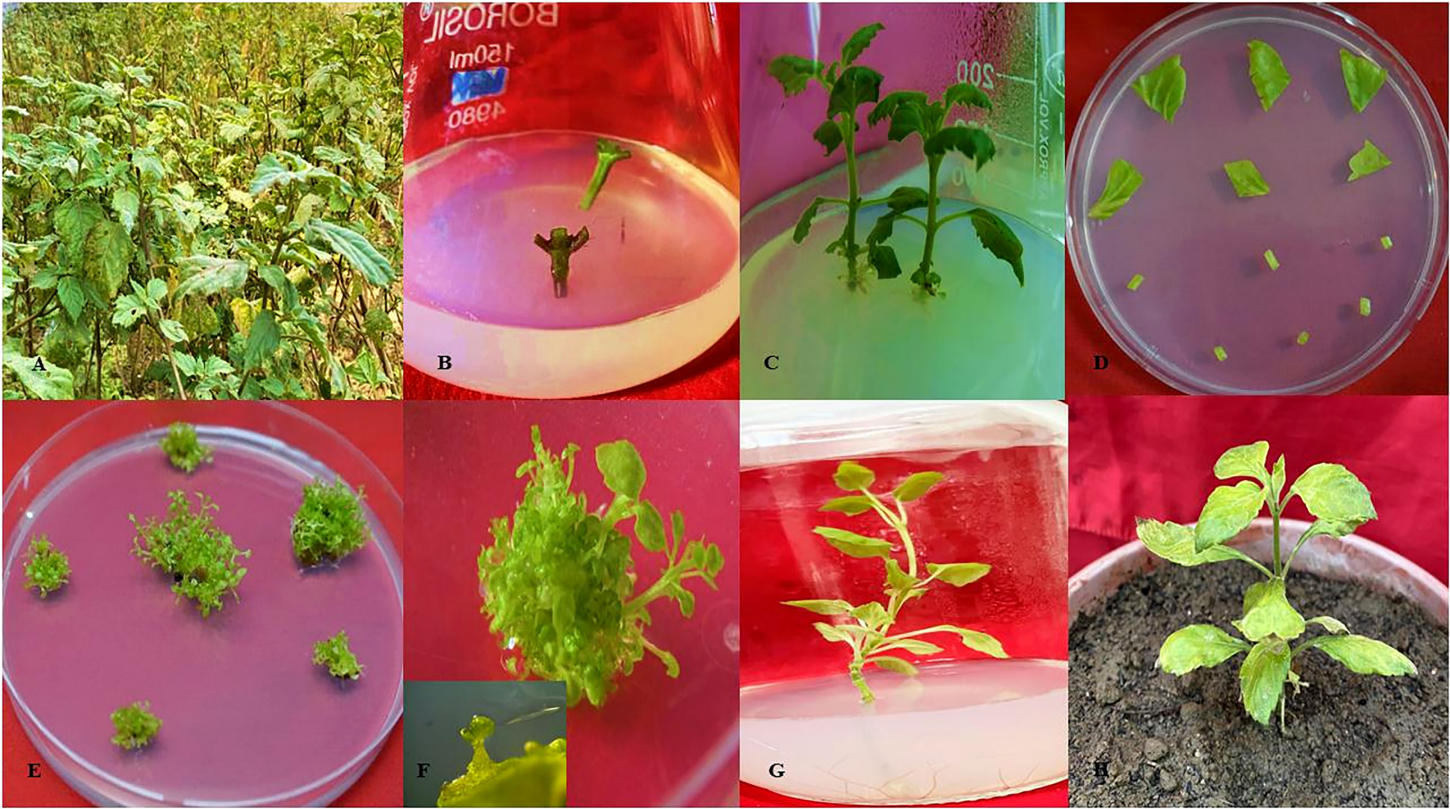
Direct regeneration of *Pogostemon cablin.* **(A)** Source of *P. cablin* plant from CIMAP experimental farm. **(B)** Nodal explants were surface sterilized and shifted to half/full strength MS medium. **(C)** *In vitro* established rooted Patchouli plants. **(D)** Leaves and petioles were on regeneration medium (MS media supplemented with 0.2 mg/L BAP + 0.1 mg/L NAA). **(E)** Direct regeneration was observed from both explants. **(F)** Multiple shoots of a single explant and its microscopic view. **(G)** Regenerated shoots were shifted on MS medium for root induction. **(H)** Rooted plants were acclimatized in the greenhouse.

Immature nodal explants were collected from 3-4 month old healthy patchouli plants (CIM-Samarth) cultivated by CSIR-CIMAP experimental farm itself, as shown in **Figure 1A**. The explants were washed with Tween20 (HiMedia, Maharashtra) and then rinsed with running tap water for 15-20 min. To sterilize the explants, they were treated with mercuric chloride (HgCl_2_, 0.1%) for 2-5 min. and then rinsed 2-3 times with sterile distilled water to remove any residual HgCl_2_. The sterilized explants were then transferred to full and half-strength MS medium (Murashige and Skoog, 1962) for *in vitro* plant establishment, as shown in **Figures 1B, C**. Further, the *in vitro* grown plants were taken as an opportunity to standardize the direct regeneration system using leaf, petiole, and internodal tTCL as explants (~0.1 to 1.0 cm in size). Eighteen explants of each type were inoculated onto MS medium containing various compositions of auxin (1-naphthaleneacetic acid- 0.01 mg/L to 1 mg/L) along with cytokinin (6-benzylaminopurine hydrochloride- 1 mg/L to 6 mg/L) for direct regeneration system (**Table 1**). The inoculated plates were maintained in the culture room at 25 ± 2°C with 16L-H: 8D-H photoperiods. After 45 days, the regenerated explants were shifted to full and half-strength MS medium for root induction (**Figure 1G**). The rooted plants were shifted to autoclaved sand for hardening, then after acclimatization plants were shifted to a mixture of soil and vermicompost (3:1) as shown in **Figure 1H**.

**Table 1:** Response of different hormonal combinations to induce direct regeneration from petiole and leaf explants.

Media composition (MS + Hormones mg/L)	Explants used			
	Petiole		Leaf	
	No. of Shoots	Regeneration frequency*(%)	No. of Shoots	Regeneration frequency*(%)
**MS**	0.0	0.00	0.0	0.00
**1.0 BAP + 0.1 NAA**	2.30 ± 0.57^b^ c^+^	10.00 ± 0.57^abc^	5.60 ± 0.80^b^ c^+^	33.30 ± 1.00^cdef^
**1.0 BAP + 0.5 NAA**	1.60 ± 0.58^ab^ c*	21.60 ± 0.58^abcd^	1.50 ± 0.54^a^ c*	10.00 ± 0.57^abc^
**1.5 BAP + 0.1 NAA**	4.60 ± 1.10^c^ c^++^	33.30 ± 1.00^cde^	1.80 ± 0.75^a^ c^++^	16.70 ± 0.90^abcd^
**1.5 BAP + 0.5 NAA**	1.60 ± 0.60^ab^ c*	26.60 ± 0.50^bcd^	0.60 ± 0.51^a^ c*	5.00 ± 0.50^ab^
**2.0 BAP + 0.1 NAA**	0.0 c+++	0	0.0 c+++	0
**0.1 BAP + 0.1 NAA**	19.30 ± 1.15^f^	71.60 ± 1.10^fg^	15.10 ± 1.16^ef^	66.70 ± 1.10^gh^
**0.2 BAP + 0.1 NAA**	33.60 ± 1.52^i^ c^-^	88.30 ± 1.12^g^	37.30 ± 1.63^i^ c^-^	93.30 ± 0.56^i^
**0.3 BAP + 0.1NAA**	27.60 ± 1.53^h^ c	76.60 ± 0.54^gf^	26.00 ± 1.78^h^ c	76.70 ± 0.55^hi^
**0.4 BAP + 0.1 NAA**	24.60 ± 1.51^g^ c^+^	55.00 ± 0.40^ef^	18.60 ± 1.50^g^ c^+^	55.00 ± 0.58^fg^
**0.5 BAP + 0.1 NAA**	19.30 ± 1.15^f^ c^++^	38.30 ± 0.52^ed^	15.00 ± 2.09^f^ c^++^	43.30 ± 0.57^ef^
**0.6 BAP + 0.1 NAA**	11.60 ± 0.50^e^ c^+++^	26.60 ± 1.20^bcd^	13.50 ± 1.20^e^ c^+++^	38.30 ± 1.15^def^
**0.7 BAP + 0.1 NAA**	8.30 ± 1.52^d^ c^+++^	16.60 ± 1.52^abcd^	10.30 ± 0.51^d^ c^+++^	38.30 ± 0.50^def^
**0.8 BAP + 0.1 NAA**	3.30 ± 0.50^bc^ c*	16.60 ± 0.49^ab^	8.50 ± 0.50^c^ c*	26.70 ± 0.53^bcde^
**0.9 BAP + 0.1 NAA**	1.60 ± 0.57^ab^ c*	5.00 ± 0.58^ab^	6.60 ± 0.81^bc^ c*	21.70 ± 0.49^abcde^

Values shows mean ± SD. Means followed by the same letters (a, b, c, d, e, f, g, h, i) within column do not differ significantly at p ≤ 0.05 according to Duncan’s multiple range test 0.0 = No response on MS medium, c-= Direct regeneration without callus, c+ = Direct regeneration with little callus, c++ = Shoot regeneration with more callus, c+++ = Maximum callus, c*= callus hard and brown.

Regeneration frequency (%) = no. of explants regenerated/ Total no. of explants inoculated x 100.

### 2.2 Histological analysis of transverse thin cell layer explants

The initial and regenerated explants were prepared for imaging under Leica Microsystem limited (Switzerland, version 2.1.0) and scanning electron microscope (SEM) to visualize histological information. Initially, explants were washed three times with deionized water to remove traces of agar, and then the transverse section was cut. The unprocessed samples were mounted on aluminum stubs using two-sided adhesive copper tape and placed in the SEM specimen chamber (FEI-Quanta 250). Images were taken in low vacuum mode using a large field detector (LFD) at a chamber pressure of 120 Pa. An accelerated voltage of 20 Kv and a working distance of 12.9 mm were used. Relatively low magnification (61X- 174X) was used to obtain the image, and the optimum spot size (4.0) was chosen to achieve better resolution.

### 2.3 Establishment of a genetic transformation using *Agrobacterium tumefaciens*

For selecting putative transgenic *Pogostemon cablin* plants containing the *ACC deaminase* gene, standardization of kanamycin concentration, acetosyringone concentration, optical density, and treatment time are prerequisites (Paul et al., 2012). Two constructs were used in this study for the genetic transformation of *P. cablin*, and both constructs were in *A. tumefaciens* strain LBA4404. One of the constructs contained a gene of interest, *ACC deaminase*, while the other was a pBI121 binary vector used as a control. The control vector carried the *gusA/uidA* reporter gene, controlled by constitutive promoter CaMV 35S and Tnos terminator. Moreover, each construct has a selection marker *nptII.*

#### 2.3.1 Optimization of Kanamycin concentration for selection of transformants

**Figure 2:**
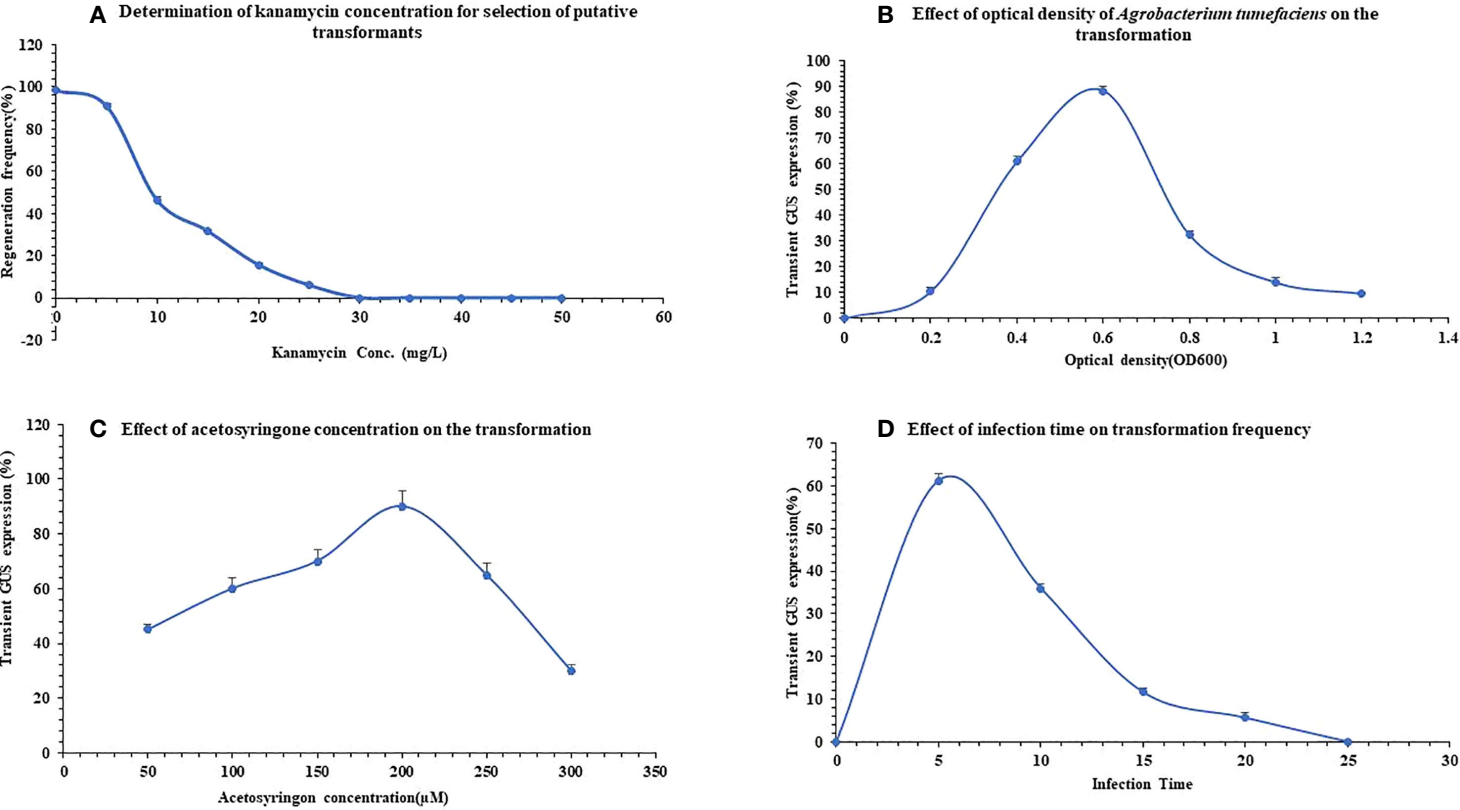
Standardization of *Agrobacterium-*mediated genetic transformation protocol in *Pogostemon cablin.* **(A)** Optimization of kanamycin concentration for selection of regenerated transformants. **(B)** Effect of *A. tumefaciens* optical density on transient *uidA* expression of *P. cablin.* **(C)** Detection of optimum acetosyringone concentration on behalf of transient GUS expression. **(D)** Evaluation of optimum infection time to get maximum transformation frequency. The bars indicate mean ± SD.

For the efficient selection of putative transformants, kanamycin concentration was optimized. Excised untransformed explants were transferred to the regeneration medium 0.2mg/L BAP and 0.1mg/L NAA containing kanamycin concentration varies from 05 to 50mg/L for screening of transformants. After 30-45 days of inoculation, plates were screened for optimized kanamycin concentration (**Figure 2A**).

#### 2.3.2 Standardization of optical density, infection time, and acetosyringone concentration

The determination of optimum optical density was also checked to obtain the maximum transformation frequency in *P. cablin.* A single colony of *A. tumefaciens* strain LBA4404 with pBI121 vector was used as the inoculum in Yeast extract broth (YEB) as per Singh et al. (2017) to get optimal OD_600._ The bacterial cells were harvested at various OD (0.2-1.2) by centrifugation at 5000rpm for 10 min. at 4° C. The pellet was then re-suspended in a liquid MS medium. All OD ranges were used to infect all explants to detect the optimal OD of bacterial strain (**Figure 2B**). To standardize infection time; explants were infected with a pBI121 vector having optimal OD_600_ 0.6 for a different time interval (5-25 min.) (**Figure 2D**). Activation of *VirA* gene is requisite to obtain the maximum *Agrobacterium-mediated* transformation frequency. Acetosyringone of different concentrations ranging from 50µM to 300µM, shown in **Figure 2C**, was added to the co-cultivation medium just before infection of the explants.

#### 2.3.3 Co-cultivation, regeneration, and root induction in transgenic plants

**Figure 3:**
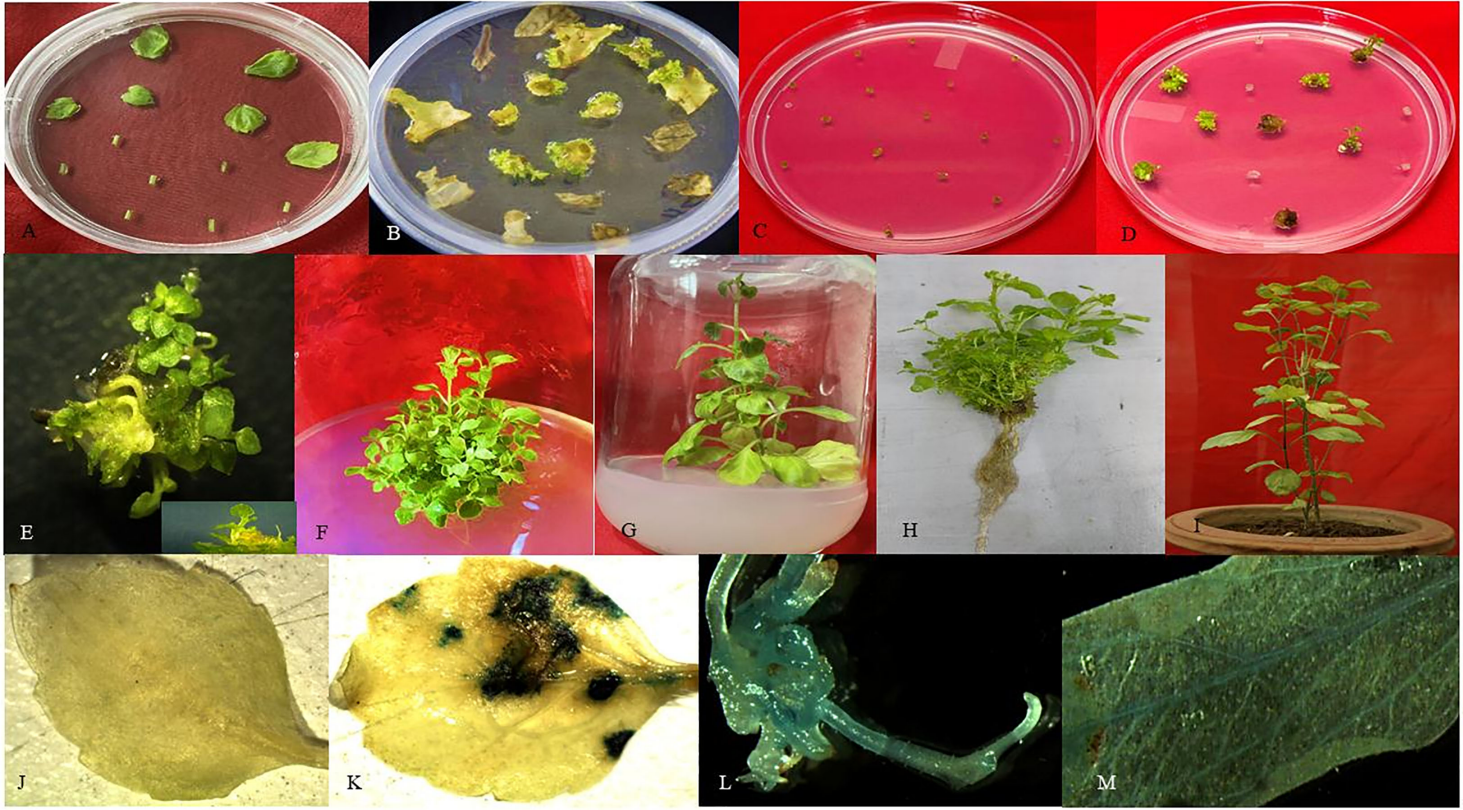
Regeneration and development of putatively transformed *P. cablin* plants. **(A, C)** *Agrobacterium-*infected explants (leaves, petioles, and internodal tTCL) were inoculated onto a regeneration medium with a selection marker. **(B)** Direct regeneration of leaves and petiole explants after 25-30 days of inoculation on selection medium. **(D)** tTCL section showed regeneration after 15-20 days on kanamycin-containing media. **(E)** Microscopic view of regenerated shoots. **(F, G)** Putative shoots were shifted to a half-strength MS medium with a selection marker for root induction. **(H, I)** Rooted putative transgenic plants were shifted in the greenhouse for hardening and acclimatization. **(J)** Non-transformed leaf (control) **(K)** Transient GUS expression of patchouli leaf. **(L, M)** Stable GUS expression of regenerated shoots and leaf of *P. cablin*.

The explants were treated with all the optimized parameters and inoculated onto MS medium for 24-72h of co-cultivation in the dark. Subsequently, the explants were shifted to a regeneration medium (as mentioned above) containing 30mg/L kanamycin and 250mg/L cefotaxime for selection of putative transformants and inhibition of *A. tumefaciens* overgrowth after co-cultivation, respectively. The regenerated putative transformants were transferred to half strength MS medium for root induction. Moreover, the rooted plants were then shifted to pots filled with a mixture of sand: soil (1:1) for hardening and acclimatization in the glass house, as depicted in **Figures 3H, I**, (Singh et al., 2017).

### 2.4 GUS histochemical assay

To validate transgenic plants, both transient and stable GUS expression was observed using the procedure described by Jefferson (1987). The leaf and petiole explants were dipped in a solution comprising 1mM X-Gluc, Thermo scientific (5-bromo-4-chloro-3-indolyl glucuronide), 0.1mM potassium ferrocyanide, 0.1mM potassium ferricyanide, 0.1M sodium phosphate buffer (pH 7.0), and 0.1% triton X-100 (HiMedia). The explants were then incubated at 37°C for 16h in the dark chamber or covered with aluminum foil (Khan et al., 2015). After incubation, the explants were washed with 70% ethanol until completely removing chlorophyll content and analyzed under the stereomicroscope for transient expression after ten days of infection, as shown in **Figure 3K**. In contrast, putative transgenic plants cultivated on the kanamycin-containing medium were checked for stable GUS expression after 8-10 weeks of infection (**Figures 3L, M**). The presence of blue color confirmed the expression of putative transgenic plants.

### 2.5 Molecular characterization of putative transgenic plants

#### 2.5.1 Polymerase chain reaction

The twelve randomly selected putative transgenic plants were evaluated for integrating *ACC deaminase* and *nptII* genes amplification through polymerase chain reaction (PCR). The plants’ genomic DNA was isolated using the CTAB method. Quantification and purification of genomic DNA were evaluated for the PCR reaction. The primer sequences used for *nptII* and *ACC deaminase* gene amplification are presented in **Table 2**. The PCR reaction (Takara master mix) program for *nptII* gene amplification was as follows: 95°C for 5 min. as initial denaturation, 35 cycles of 95°C for the 30s as secondary denaturation, 53°C for 40s as annealing, and 72°C for 1 min. as elongation, and 72°C for 10 min. as a final extension. Subsequently, *ACC deaminase* amplification was performed, as reported by (Singh et al., 2021). The reaction performed for 25 µl reaction (50ng DNA, 10 pmole primers, 2X PCR buffer, and 1.25 unit of Taq DNA Polymerase, Takara) at 94°C for 5 min. as primary denaturation, 35 following cycles of 94°C for the 30s as secondary denaturation, 57°C for 30s as annealing, 72°C for 2 min. as elongation, and 72°C for 5 min. as a final extension. Both amplified products, *nptII*, and *ACC deaminase*, were analyzed in 0.8% (w/v) agarose gel prepared in 1X TAE buffer in the presence of 6 µl/100ml EtBr.

**Table 2:** list of primers used in PCR and RT-PCR.

Gene	Primer sequence (from 5’ - 3’)	Accession number
*nptII*	F-AAGATGGATTGCACGCAGGT R-TCAGAAGAACTCGTCAAGAAGGC	AF485783
*PcACCD*	F- ATGGATCTGCAACGCTTTCCCC R- TTATCCGTTGCGGTAGAG	WP_013392321
*PcActin*	F- TCCCTCATGCAATCCTTCGT R- CCTCACAATTTCCCGCTCTG	KP676600
*PcACCD* (RT-PCR)	F- CTATTCGGACGCGGTCTACG R- GAAGCCGATGTCGAAACCCT	WP_013392321

F - Forward, R - Reverse.

#### 2.5.2 Gene expression analysis through real-time PCR

The relative expression of the *ACC deaminase* gene in PCR-positive lines was confirmed through RT-PCR. Total RNA was isolated from 7 transformed lines and one non-transformed plant (negative control) using TRI reagent^®^ (Sigma). Quantification was performed by Nanodrop spectrophotometer ND1000. The cDNA was prepared using 5µg of total isolated RNA with Gene Sure First strand cDNA synthesis kit (Pure gene). RT-PCR-specific Primers for *ACC deaminase* and *Actin* (endogenous control) were designed using Primer3 software and are listed in **Table 2**. To perform RT-PCR, cDNA was diluted to 100-150 ng/µl, and 5pmol of forward and reverse primers were used. Moreover, the target gene’s relative expression was calculated by the 2-^ΔΔCt^ method (Afroz et. al., 2022), and normalization of the target gene was done using the comparative Ct value of endogenous control.

### 2.6 Structure modeling and molecular docking

Currently, computational studies have been used extensively to understand the ligand-receptor interaction between ACC and *ACC deaminase* to mitigate abiotic stress (Suresh et al., 2022). The 3D structure was deduced by depositing *ACC deaminase* (*Achromobacter xylosoxidance*) primary protein sequence from the NCBI database (http://www.ncbi.nlm.nih.gov) (WP_013392321.1) in SWISS-MODEL (http://swissmodel.expasy.org/workspace). Further, the result was narrowed to get a representative model 1F2D for homology modeling, which has >98% query coverage and >59% identity. The best preliminary predicted model was processed for refinement by Galaxy refine (http://galaxy.seoklab.org/) server. Stereo-chemical properties of the build model were further analyzed using the PROCHECK tool in the SAVES server (http://nihserver.mbi.ucla.edu/SAVES/) based on the Ramachandran plot to know the quality of the built model (Laskowski et al., 1993). The model was further visualized by PyMOL (DeLano, 2002).

**Figure 4:**
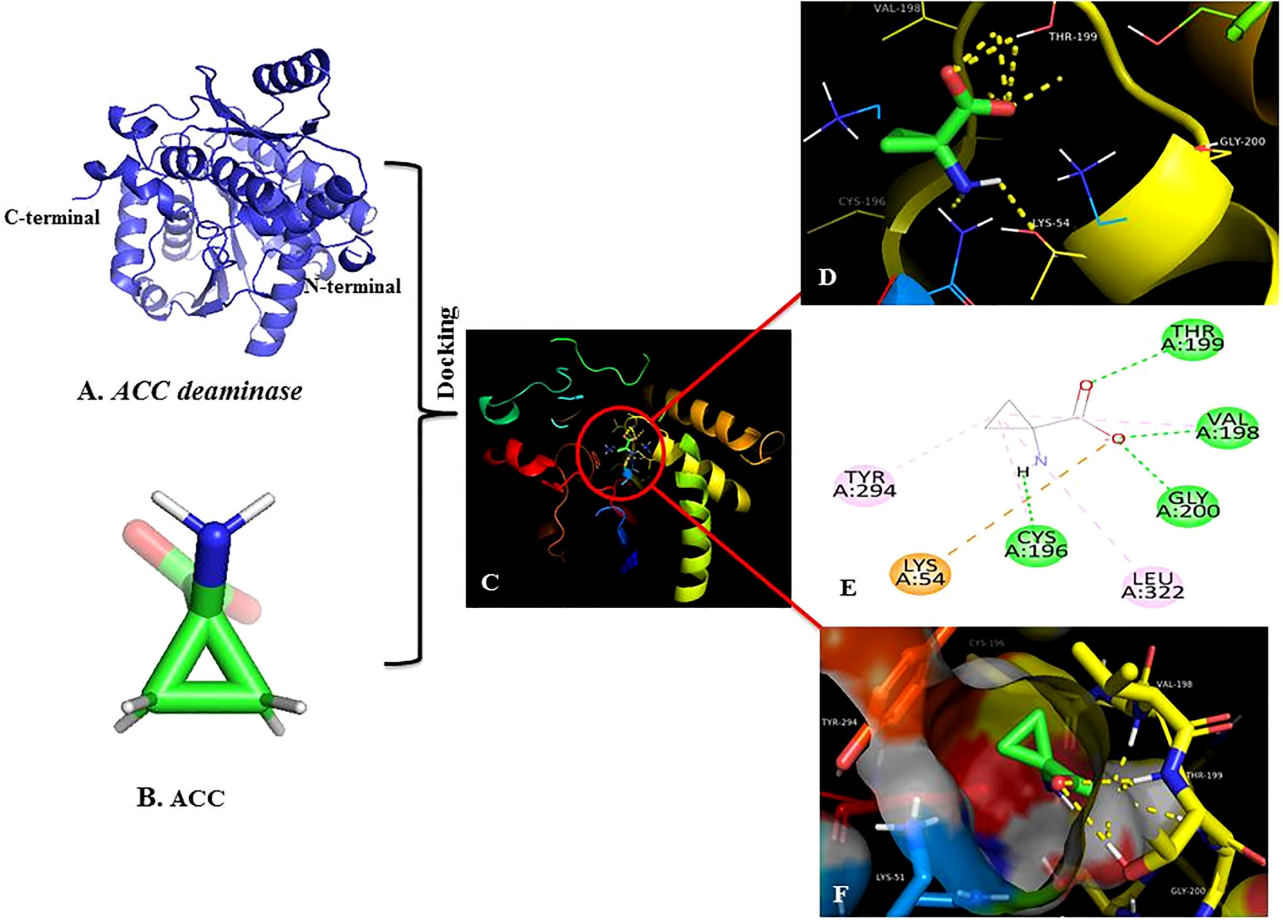
Homology modeling and molecular docking. **(A)** 3D homology model of receptor ACC deaminase. **(B)** ACC ligand molecule **(C)** Molecular docking of receptor and ligand at the active site of target protein ACC deaminase. **(D)** PyMOL pictorial representation showed H-bonds interaction at the catalytic subunit. **(E)** 2D Docking- showed interaction of the ligand with amino acid present at the active site of target protein, green dots indicating conventional H-bonds. **(F)** Docking stable conformation at the binding pocket between ACC and ACC deaminase.

Molecular docking was performed using AutoDock tool version 4.2.6 (Perkin Elmer, Massachusetts, USA). The modeled structure was docked with ligand ACC (**Figure 4B**) (Compound CID: 535). Grid Box was modified to cover the binding pocket of *ACC deaminase* at center X = 35.964, center Y= 118.074, center Z= 15.704, and the number of points in all dimensions was 60A°. Genetic algorithm simulation was performed for 50 independent docked calculations. The docked conformation with the lowest energy was visualized by PyMOL version 2.4.0 and discovery studio version 21.1 to study interactions in active sites binding pockets (DeLano, 2002).

### 2.7 Drought stress treatments

To investigate the response of *ACC deaminase* expression, 2-3 months old 12 transgenic lines (PT4) and 12 non-transformed plants were selected to undergo drought stress in the glasshouse control environment. Three different watering conditions were prepared as per Yamasaki and Dillenburg (1999). Different setups were planned, i.e., sufficient water (15% soil moisture, 100ml distilled water on alternate days), modest water (12% soil moisture, 100ml distilled water on every fifth day), and extreme water stress (8% soil moisture, no water given until day20). Well-watered treatment was used as control. On the day 21 of the dehydration experiment, samples were collected for various morphological and physiological analyses.

#### 2.7.1 Assessment of relative water and chlorophyll content

The third leaf of each plant (Control and transgenic) was used for analysis. To get relative water content (RWC), the fresh weight (FW) of leaves was taken. Afterward, the leaves were kept in water for 180 min. to obtain turgid weight (TW). The turgid leaves were then kept in the oven at 42°C for 30h, and dry weight (DW) was taken. The RWC was calculated by the formula RWC (%) = [(FW-DW)/(TW-DW)] X 100.

To calculate the amount of chlorophyll a and b, 100 mg of powder (crushed leaves in liq. N_2_) were kept overnight at 4°C in 10 ml of 80% acetone. The next day, samples of drought stress were centrifuged at 5000rpm, 4°C for 15 min. Then the supernatant was collected, and absorbance was checked at A_663_ and A_645_nm by using a spectrophotometer (Multiskan™ Go, Thermo Scientific) to measure chlorophylls as described by Arnon (1949).

#### 2.7.2 Quantification of reactive oxygen species production

**Figure 5:**
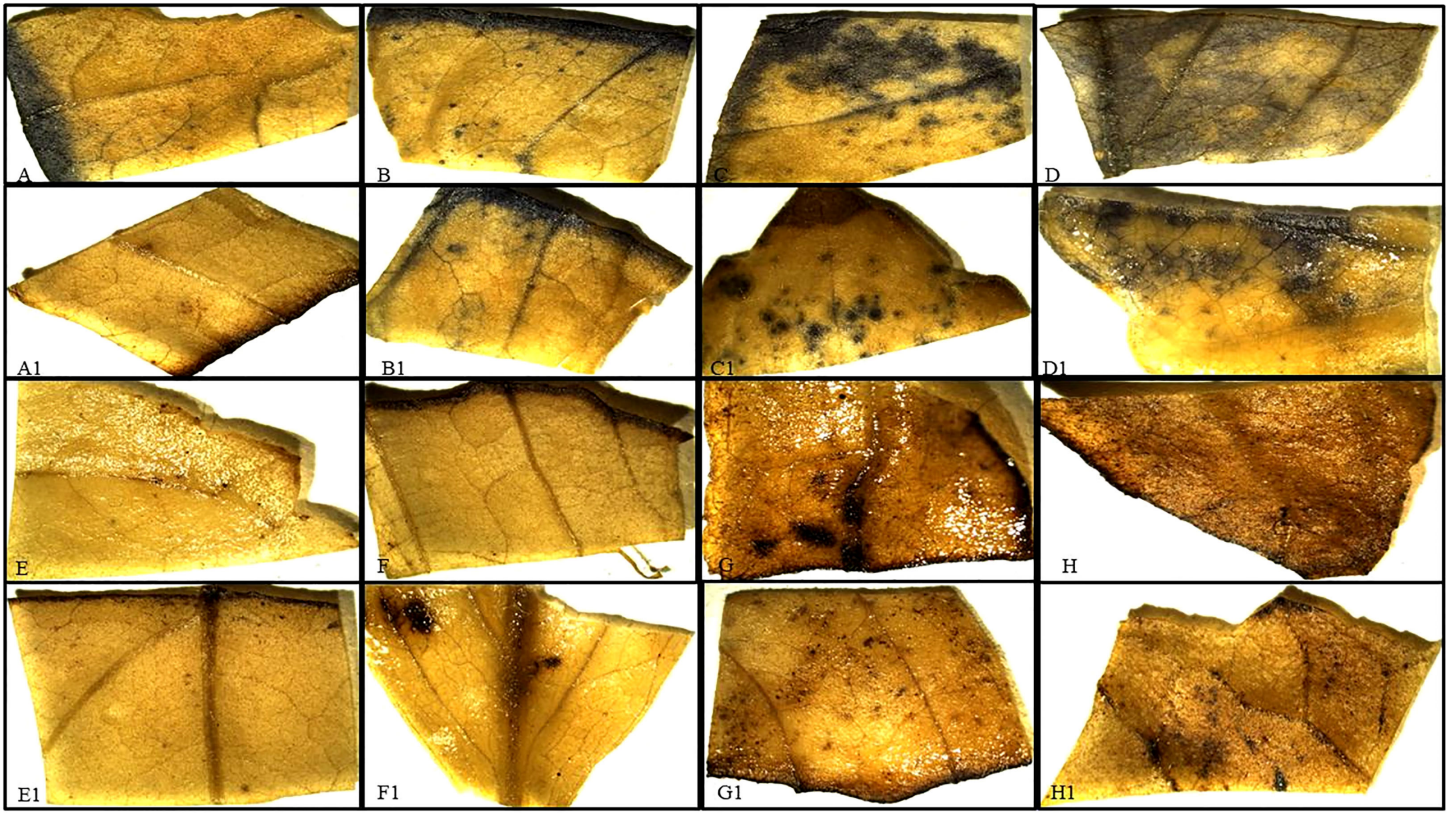
Evaluation of ROS production in transgenic plants under drought stress **(A-D)** NBT stained wild-type patchouli leaves, indicating as the severity of dehydration stress has increased from 15-8% of soil moisture in pots; blue dots increased which represent amplified superoxide, as compared to **(A1-D1)** transgenic *P. cablin* leaves **(E-H)** DAB-stained leaves showed with increasing drought stress accumulation of H_2_O_2_ (brown spots) has enhanced in wild type as compared to **(E1-H1)** transgenic patchouli leaves.

The severity of stress in treated and non-treated plants can be achieved by calculating the hydrogen peroxide production rate assay. To perform 100mg of leaf samples grounded in 0.1% Tri-chloroacetic acid (TCA) followed by centrifugation at 12000rpm, 20 min., 4°C. Further, a mixture of 400 µl supernatant, 400 µl of 10mM phosphate buffer (pH -7.2), and 800 µl of Potassium iodide was used to estimate absorbance at 390nm and compared with the standard curve of H_2_O_2_ (Singh et al., 2021). Moreover, hydrogen peroxide and superoxide were visualized on tested leaves by using 1mg/ml solution of 3, 3’-Diaminobenzidine (DAB) (pH-3.8) and nitro blue tetrazolium (NBT), respectively. Leaves in both solutions were kept overnight in light, and stained leaves were washed 3-4 times with 70% ethanol to remove chlorophyll content. Observe brown and blue spots in **Figure 5** that appear due to the production of H_2_O_2_ and O_2_^-^ respectively. Further, data were correlated with literature to evaluate drought tolerance rate in transgenic plants compared to control.

### 2.8 Data analysis

All experiments were carried out in three biological replicates, and data were shown as mean ± S.D. Statistical studies were conducted using one-way ANOVA to analyze significant differences between means using IBM SPSS statistics 29.0 (SPSS Inc. USA). The one-way variance was performed using Duncan’s multiple range test at a significant value of p<0.05 (Duncan, 1955).

## 3 Results

### 3.1 Establishment of Direct regeneration system from petiole explants

In the present research leaf, petiole, and tTCL were used as explants for direct regeneration (**Figure 1D**) and compared for regenerative potential under various growth regulator treatments (**Table 1**). Different plant growth regulators (BAP and NAA) were used to supplement the MS medium for optimization of regeneration frequency in *Pogostemon cablin*. As per the data analysis, the increment in BAP concentrations from 0.1 to 1.0 mg/L showed that no. of shoots were gradually increasing. Subsequently, the role of NAA in different combinations with BAP was analyzed, and it is concluded that the addition of NAA increased no. of shoots per explant to 0.1 mg/L, further enhancement of NAA, leading to callus formation. **Table 1** shows that maximum regeneration frequency was 88.30 ± 1.12%, 93.30 ± 0.56% per explant acquired from petiole and leaf, respectively, on MS medium supplemented with 0.2 mg/L BAP and 0.1 mg/L NAA without callus (**Figures 1E, F**). A comparative study revealed that the best direct shoot organogenesis observed from leaf explants which were 93.30 ± 0.56%. As per **Figure 1G** regenerated shoots with 6-8 leaves were shifted on a half-strength MS medium for root induction. Further rooted plantlets were shifted to pots containing a mixture of soil: vermicompost (3:1) for acclimatization in greenhouse conditions, showing a survival frequency of 66.0 ± 0.6% (**Figure 1H**).

### 3.2 Histological analysis of tTCL

**Figure 6:**
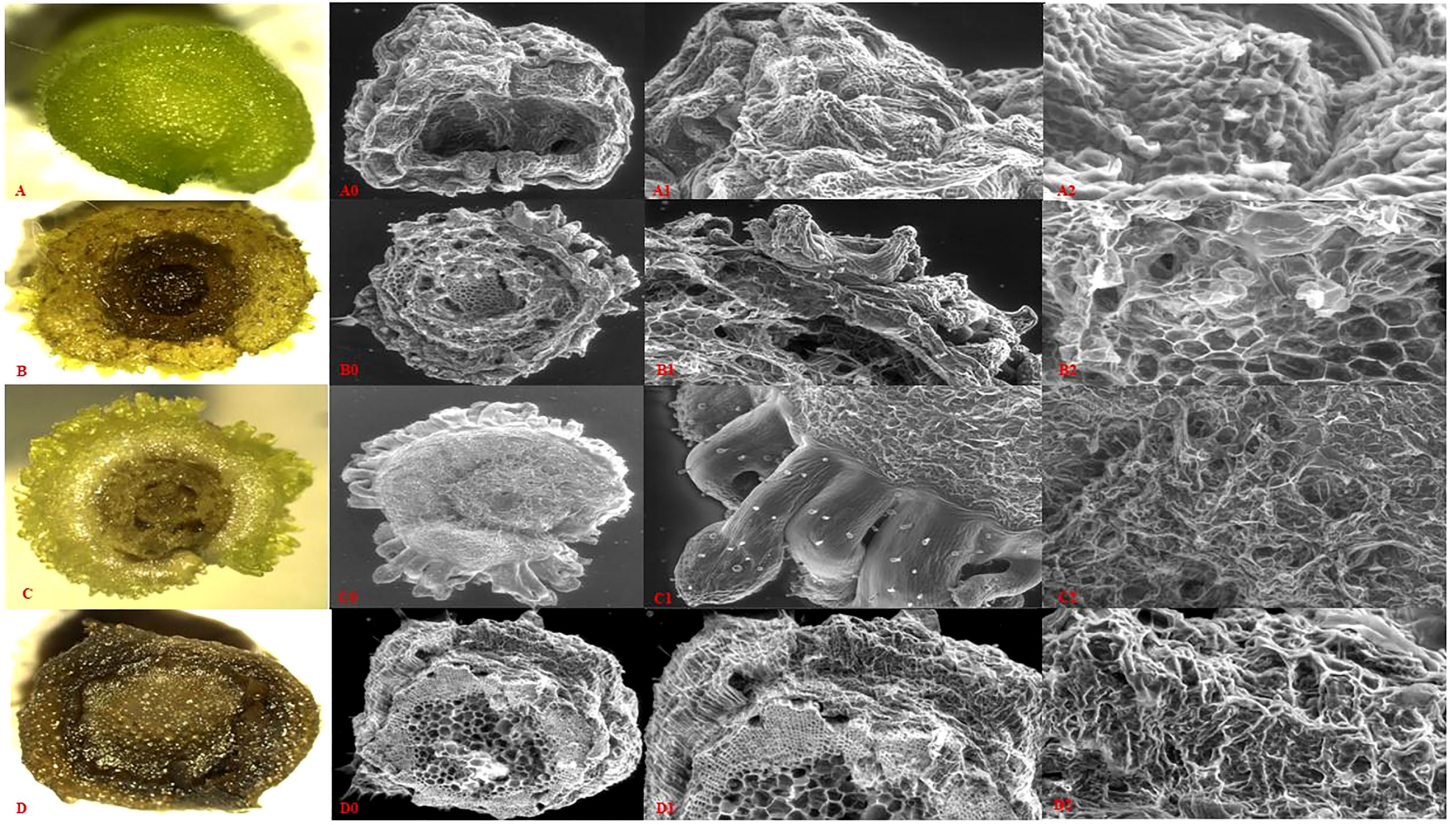
Microanatomy of *P. cablin*, tTCL internodal section and its direct regeneration at different stages on regeneration medium. **(A-D)** Microscopic view of tTCL section at different stages (0, 10, 15, and dead cells) of direct regeneration. **(A0-D0)** Scanning electron microscopic view of tTCL section of above mention stages. **(A1-D1)** SEM of the initially induced organogenesis with different time intervals (0, 10, 15 days - A1, B1, and C1 respectively) at 61x magnification and D1 showed dead cells at the same magnification. **(A2–D2)** Different developmental stages of emerging multiple shoot buds at 174x magnification.

The microanatomy information of transverse thin cell layer section of different stages (0, 5, 10, and 15 days) showed that as the cells move from initial to 5 days, cell size gradually changes from meristematic cells, different from nearby cells. Regeneration of cells was visualized at 10 days of inoculation from vascular cells, and subsequently, shoot buds were visualized at 15 days of inoculation (**Figures 6B, C**) as compared to the initial stage of the tTCL section (**Figure 6A**). As per analysis optimum regeneration was faster (15-20 days) than conventional explants (25-35 days). Further, multiple shoot meristem were developed into *in-vitro* plants.

### 3.3 Optimization of Kanamycin concentration for selection of putative transformants

Obtained data were analyzed based on inhibition of regeneration frequency, shoot number, and necrosis of shoot to optimize kanamycin concentration. Explants inoculated on a regeneration medium containing 5mg/L antibiotic dose showed maximum regeneration frequency (91.0 ± 0.05%) whereas, further increments in antibiotic concentration gradually decreased the regeneration. At 30mg/L, no regeneration from the explant was obtained (**Figure 2A**) whereas at 25mg/L few shoots buds were regenerated and most of them were gradually bleached after 3-4 weeks on the selection medium. Above 30mg/L, kanamycin indicated inhibition of shoot regeneration. These observations concluded that 30mg/L kanamycin concentration was optimum for selecting putatively transformed patchouli shoots on regeneration medium.

### 3.4 Evaluation of *Agrobacterium tumefaciens* optical density and infection time on transformation

The efficacy of bacterial OD was checked at various OD ranges (0.2-1.2) to get maximum transformation frequency. With the increase in (OD_600_) from 0.2 to 0.6, the efficacy of transient GUS expression was enhanced from 10.6 ± 0.8% to 88.3 ± 0.8% (**Figure 2B**) with fewer brownish explants. Earlier reports convey similar results where optimal transformation frequency was achieved from cell density 0.6-0.8 (Ribas et al., 2011; Singh et al., 2017). Further increment in optical cell density till 1.0 showed a decline in transient GUS expression, which indicate a decrease in transformation frequency (14.0 ± 1.0%) and 83.0 ± 0.1% explants turned brown.

The effect of infection time with *Agrobacterium* is another essential factor in the transformation efficiency. Hence, a range of time periods of 5-25 min. was assessed. As the time period increased from 0 to 5 minutes, transient GUS expression was increased and reached its maximum at 5 min. of incubation 61.3 ± 0.7%, as presented in **Figure 2D**. However, continuous increment in infection time leads to a decline in transformation frequency 5.6 ± 0.6% at 20 min. (**Figure 2D**). Earlier, Paul et al. (2012) analyzed that longer infection time can reduce transient GUS expression due to the death of explants.

### 3.5 Impact of acetosyringone concentration on transformation frequency

To enhance *Agrobacterium-*mediated transformation frequency, various acetosyringone (AS) concentration of 50µM-300µM was used in an *Agrobacterium* co-cultivation medium with pricking as an injury making explants more susceptible to infection. After a few days of infection, transient GUS expression of explants was analyzed that showed a gradual increment from 41.3 ± 0.5 to 89.3 ± 0.9 on the addition of AS from 50µM to 200µM respectively. As acetosyringone concentration was increased further from 200µM to 300µM, transient GUS expression declined to 30.3 ± 0.7 (**Figure 2C**). This result concluded that 200µM AS concentration is best for achieving maximum transformation frequency in *P. cablin.*

### 3.6 Regeneration and development of putative transformed explant

Transformation with *Agrobacterium* strain vector containing *ACC deaminase* and control was performed as per standardized protocol. Further, Putative transformed explants were inoculated after co-cultivation on regeneration media with optimized 30mg/L kanamycin and 250mg/L cefotaxime concentration for regeneration and allowed to regenerate for 25-35 days. Leaf and petiole explants took 25-35 days to regenerate, whereas tTCL explants took only 15-20 days (**Figures 3A–E**). Regeneration frequency was 46.10 ± 0.35% onto regeneration medium supplemented with kanamycin. Subsequently, regenerated shoots were subcultured five times to remove the chimera. Putative transformed shoots were placed onto root induction media supplemented with kanamycin for approximately 8-10 weeks to get rooted plants and shifted to the greenhouse for hardening and acclimatization (**Figures 3F–I**). During the hardening procedure survival rate of healthy rooted plants was 52.0 ± 0.8%.

### 3.7 GUS assay

Transient GUS Expression was analyzed after 5-6 days of infected explants, i.e., optimized infection time 15 min, co-cultivation, AS concentration 200µM, and OD_600_ 0.5. GUS positive putative transformed explants were showing blue color. Moreover, stable *gusA* expression was also analyzed for transformed and non-transformed plants under the microscope after 8- 10 weeks. As illustrated in **Figures 3J–M**, leaves and petioles were blue.

### 3.8 Molecular Analysis of Transformed *Pogostemon cablin* Plants

#### 3.8.1 PCR and RT-PCR amplification

**Figure 7:**
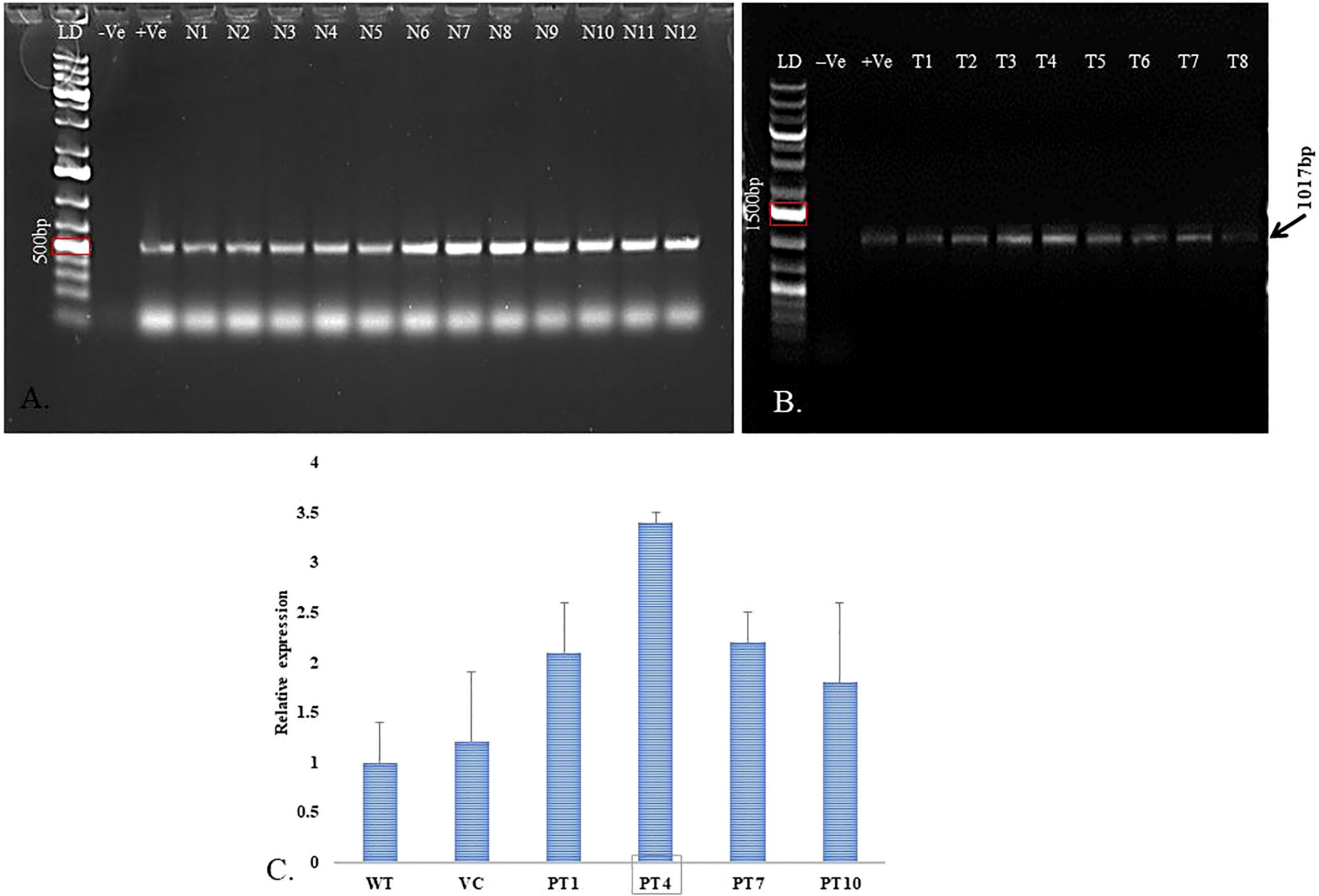
Molecular analysis of transformed *P. cablin* plants. **(A)** Genomic DNA was used to verify the presence of selection marker *nptII* gene (500bp PCR product, LD- 1kb+ ladder; - Ve –negative control; +Ve –positive control; N1-N12 are *nptII* positive transgenic lines). **(B)** Healthy transgenic lines were screened for *ACC deaminase* gene-specific PCR amplification to obtain 1017bp product on 1% agarose gel (LD- 1kb+ ladder; -Ve –negative control; +Ve –positive control; T1-T8 are *ACC deaminase* +ve transgenic lines). **(C)** RT-PCR analysis for relative expression of *ACC deaminase* gene in different transgenic *P. cablin* lines and *actin* gene was used for normalization of the template (WT- Wild type; VC- Vector control; PT- Patchouli transgenic lines). The bars indicate mean ± SD.

The selection marker *nptII* gene-specific PCR product was analyzed through gel electrophoresis that showed the presence of the expected size of a single band (500bp) in **Figure 7A**. Further, a few healthy lines were analyzed for *ACC deaminase* gene integration into the genome of patchouli. The PCR products confirmed the integration of ‘*ACCD*’ (1017bp) in the above-mentioned line as presented in **Figure 7B**. Genomic DNA of wild-type patchouli plant was used as a negative control, and vector construct was used as the positive control. Quantitative PCR analysis showed actin and ACC deaminase gene transcripts in four of the PCR-confirmed transformed plants. Moreover, **Figure 7C** showed that line T4 has maximum relative expression.

### 3.9 Homology modeling and docking validation

**Figure 8:**
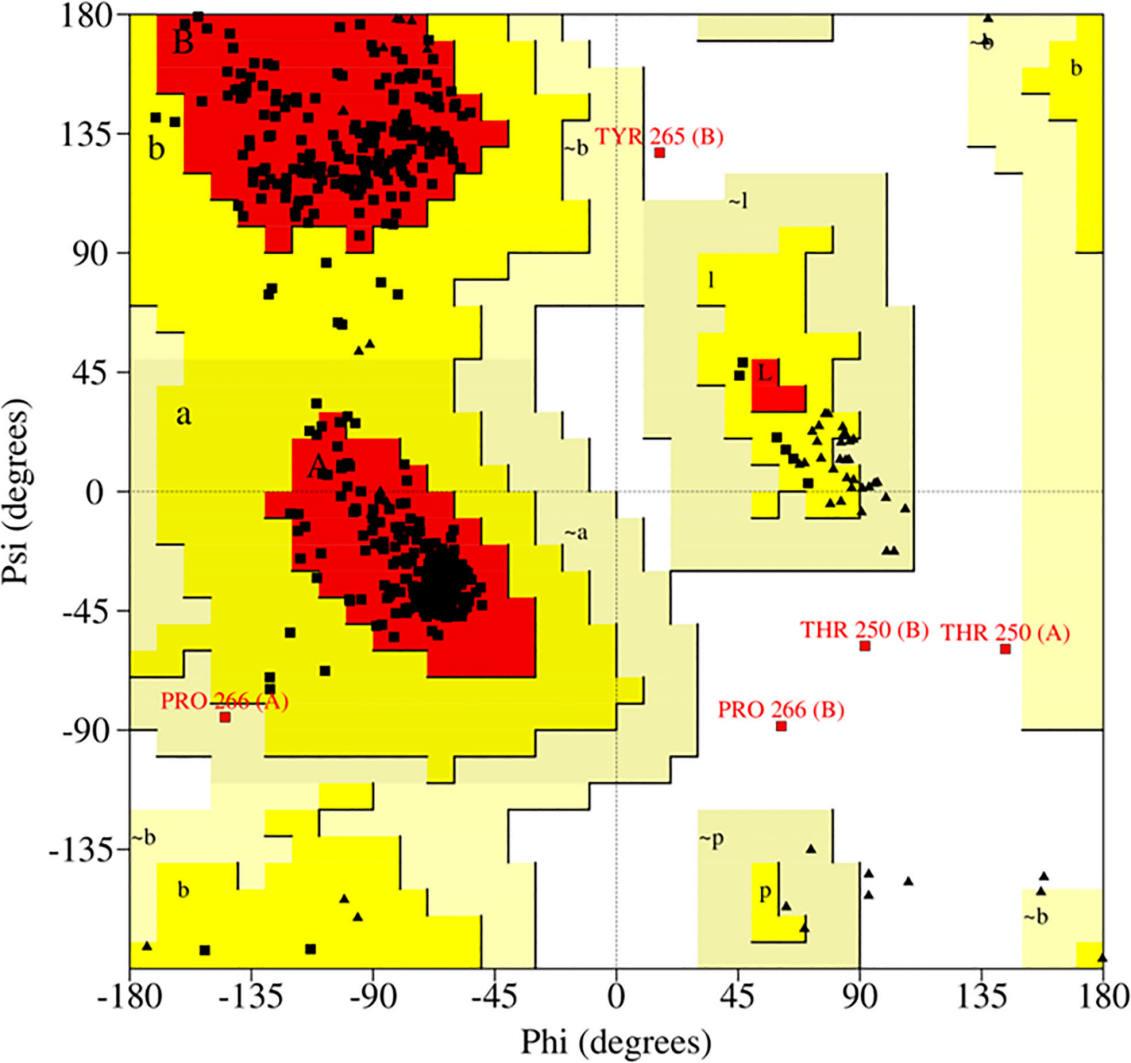
The 3D crystal structure of ACC deaminase was validated by using the online server PROCHECK. According to the Ramachandran plot, only 0.5% of residues fall in the disallowed region and 99.5% of residues come under the allowed region.

Successful *in-vitro* results were worth performing homology modeling and molecular docking studies. The 3D model of the target enzyme was prepared in **Figure 4A**, using SWISS-MODEL, by considering the best match ‘1f2d’ as a template (Schwede et al., 2003). The model protein was validated using different online servers, and quality was evaluated by Ramachandran plot of *ACC deaminase* (**Figure 8**) showed 94.6% (539) of amino acid residues resided in the most favorable region. The residue falling in generously favourable regions was 4.9% (28), and no residues 0.0% (0) were in additional favour, while only 0.5% (3) of residues were in unfavourable regions. As per Lovell (2003), these data were expected statistics. Afterward, the validated protein model was considered to carry out the docking procedure using the Autodock tool. Multiple docking poses were analyzed for optimal ligand-receptor complex, indicating the best scoring and lowest binding energy to get maximum binding affinity. Further best confirmation was used to visualize in discovery studio, and PyMOL for ligand interaction showed four hydrogen bonds between ACC and surrounding amino acids (Cys-196, Val-198, Thr-199, and Gly-200), indicating high binding affinity with receptor shown in **Figures 4C–F**.

### 3.10 *PcACC deaminase* heterologous expression enhanced drought stress tolerance

#### 3.10.1 Estimation of RWC and Chlorophyll content in transgenic lines

**Figure 9:**
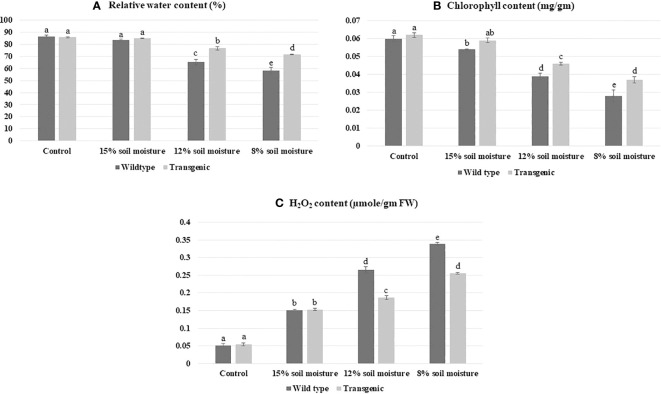
Validation of enhanced drought stress tolerance in transgenic patchouli plants, **(A)** at day 21 of the experiment, pots with only 8% soil moisture retained RWC 71.7 ± 2.3% to 75.7 ± 2.1% which is higher as compared to the RWC of control 58.3 ± 0.21%. **(B)** With the increase in water scarcity, the total chlorophyll content of control dropped to ~ 2.3 fold, although the transgenic line showed ~1.6 fold decrease as compared to the control. **(C)** WT showed a higher accumulation of H_2_O_2_ ~6.6 fold than transgenic plants ~5 fold. All experiments were performed in three biological replicates. Bars showed mean ± SD, statistical analysis was carried out using one-way ANOVA using Duncan’s multiple range test at a significance value of p<0.05.

RWC is an indicator of plant water retention capacity; therefore, it works as an experiment to analyze drought stress tolerance (Naing et al., 2021). On the day 21 of the experiment, the RWC of pots having 15% soil moisture was 83.5 ± 0.4% in control and 84.9 ± 1.4% in transgenic one as compared to pots having 12% soil moisture (65.4 ± 2.3% control and 76.6 ± 3.8% Transgenic line). Whereas, transgenic plants had better RWC than control plants in modestly watered stress conditions. The pots containing only 8% soil moisture had an RWC of 71.7 ± 2.3% to 75.7 ± 2.1%, more significant than the RWC of the control plant, 58.30 ± 0.21% at severe stress conditions (**Figure 9A**).

Chlorophyll, a photosynthetic pigment has a significant role in the absorption of light energy during photosynthesis; therefore, any variation in chlorophyll content due to stress leads to a change in the photosynthetic system of plants. Under drought stress, the total chlorophyll content of control and transgenic lines showed a ~1.5-fold decrease in 12% soil moisture condition as compared to sufficient water treatment (used as control) in **Figure 9B**. As the severity of drought increased (8% soil moisture) chlorophyll content lessened to ~2.14-fold in wild type as compared to wild control plants. In contrast, the transgenic line showed a ~1.67-fold decrement in chlorophyll content as correlated to transgenic control. (**Figure 9B**). The finding evaluates, *P. cablin* transgenic plants have ~1.32-fold higher chlorophyll content in contrast to wild type to combat the drought stress under severe stress conditions.

#### 3.10.2 Estimation of ROS (H_2_O_2_ and O_2_^-^) production under dehydration treatment

Analysis of H_2_O_2_ storage in plants was observed and found that as scarcity of water increases, ROS concentration elevated to ~6.6 fold in control and ~5 fold in transgenic lines as compared to well-watered plants (**Figure 9C**). DAB, used for histochemical investigation of *P. cablin* leaves revealed that *ACC deaminase* expressing transgenic lines were showing less amplified brown spots as compared to wild type under severe stress (**Figures 5E–H, E1–H1**). Another ROS (O_2_^-^) accumulation was detected by NBT solution by observing various ranges of blue spots based on soil moisture content in both wild and transgenic plants (**Figures 5A-D, A1-D1**). The result showed that transgenic lines expressing heterologous genes generate less O_2_ than the wild type, which combines with NBT and produces insoluble blue formazan.

## 4 Discussion

The commercial demand for patchouli has been increasing due to its tremendous aromatic and medicinal properties. However, the supply of patchouli oil, to meet this global demand, is mainly provided by Indonesia, which massively cultivates versatile patchouli varieties. *P. cablin* cultivars face drought stress while maintaining healthy patchouli plants, which limits its propagation rate. In this study, a transgenic patchouli drought-resistant variety has been developed that keeps the promise of maintaining propagation rate and yield under drought stress. For the development of transgenic plants an efficient and quick regeneration system was required hence a comprehensive analysis on the basis of culture media, explants, and the effect of different phytohormones was performed (Roychowdhury and Tah, 2011; Roychowdhury et al., 2012; Reddy et al., 2013). During this study plant growth hormone was optimized for better regeneration and explants were compared to achieve a higher multiplication rate under sterile conditions. In our findings, maximum direct shoot organogenesis was obtained from leaf explants on MS medium supplemented with 0.2 mg/L BAP and 0.1 mg/L NAA without callus (**Figures 1E, F**). As per the literature, *P. cablin is* commonly propagated through leaf discs like most herbaceous plants (Paul et al., 2010; Swamy and Sinniah, 2016). An analysis of previously documented studies, MS medium supplemented with BAP showed considerable efficacy in shoot regeneration but much less than meta-topolin (Lalthafamkimi et al., 2021; Kumaraswamy and Anuradha, 2010; Kukreja et al., 1990; Jin et al., 2014). Sun et al. (2009) have also reported that cytokinins are known for shoot organogenesis. However, Sales and Butardo (2014) revealed that PGRs BAP and NAA are significant causes of clonal variability among micro-propagated plants. The presence of NAA combination with BAP in MS medium has been reported to improve shoot induction frequency from petiole explant in *Pelargonium graveolens* (Singh et al., 2017) and *Mentha piperita* (Sarwar et al., 2009).

Van (1973) has introduced the thin cell layer (TCL) technique which is an economical, rapid, and thoroughly reproducible *in-vitro* propagation method for the up-scale production of genetically stable plants. The recent study is the first contribution to support *in vitro* multiplication of *P. cablin* using tTCL explants. The histological analysis of direct shoot regeneration using tTCL was validated to hold the strategy of rapid propagation (15-20 days) as compared to other explants (25-35 days) on a more suitable MS medium supplemented with 0.2 mg/L BAP and 0.1 mg/L NAA (**Figures 6B, C**) and half-strength MS medium was found to be efficient for rooting. Previously, as per Tripathi et al. (2018) report, it was observed that vascular zone cells have a high tendency to generate new shoot buds and the regeneration capacity of explants depends on nutrient transport across media to tTCL, which has more exposed cells on media as compared to conventional large-size explants. Similar studies have also been reported in *Talinum triangulare*, *Bacteris gasipaes*, and *Dendrobium candidum* (Steinmacher et al., 2007; Zhao et al., 2007; Swarna and Ravindhran, 2013). According to the literature, TCL explants have a more significant reproducing tendency than conventional explants (Da Silva and Dobránszki, 2015). Moreover, Raomai et al. (2015) observed that the developed rhizomes of endangered *Paris polyphylla* using tTCL basal stem explant showed significant enhancement of secondary metabolite production and mass propagation system.

Recent advancement in R&D technologies provides insight into microbial-plant interactions that help us to enhance crop yield and quality through vast biotechnological approaches such as genetic manipulation (Mamgain et al., 2013; Roychowdhury et al., 2013; Khan et al., 2014; Pathak et al., 2022). In a drought state, the plant produces stress ethylene that causes various physiological and metabolic damages in plants (Zhang et al., 2018). Literature confirmed that *ACC deaminase* has the capacity to reduce stress ethylene levels under abiotic stresses (Penrose and Glick, 2003) by degrading ACC into α-ketobutyrate and ammonia (Li et al., 2019). Generally, for the production of putative transgenic *P. cablin* plants having *ACC deaminase* gene, *Agrobacterium-*mediated genetic transformation protocol establishment is required for the highest transformation frequency. The present study revealed OD_600_ 0.6, AS 200µM, 30mg/L kanamycin, and an infection time of 5 min. is optimum to achieve the maximum transformation rate (**Figures 2A–D**). Kanamycin, used as a selection marker, has been well studied to optimize its concentration for versatile crops. Earlier Paul et al. (2012) has been reported a lower 20mg/L and Sugimura et al. (2005) reported a higher 100mg/L concentration of kanamycin for indirect regenerated putative transformed shoot selection.

Hence, the plant has innate natural resistance against kanamycin; therefore, sensitivity towards kanamycin varies among plant tissues and species of plants (Colby and Meredith, 1990). In literature, other parameters such as bacterial optical density and infection time were studied for optimum heterologous transgene expression. Dutt and Grosser (2009) recommended maximum transformation frequency on lower optical cell density. Moreover, several studies have also suggested that higher OD_600_ values reduce transformation frequency (Saini and Jaiwal, 2007). In addition, similar studies were shown better transformation frequency in less than 30 min. in other plants (Khan et al., 2015; Benazir et al., 2013), unlike (Gupta and Rahman, 2015; Singh et al., 2017). From these observations, we can conclude that variation in infection time to get maximum transformation frequency depends on tissues, species, and plants.

Stachel et al. (1985), reported that AS is a potent phenolic signaling molecule that is generally secreted by wounded plant tissues and helps in the transfer of T-DNA from Ti- plasmid by inducing a signaling cascade of all *Vir* genes after binding to the *VirA* protein of bacterial cell. Though every plant has less than the threshold value of endogenous phenolic molecules for efficient transformation, the external addition of AS in the co-cultivation medium fulfills the demand for signaling molecules to obtain optimum transformation frequency. Our study reported 200µM AS limit was best suited for transgenic development whereas in other reports 150µM of AS is optimum for patchouli leaf transformation (Paul et al., 2012), Adverse effect on transformation frequency of plants has also been reported in other species with an increment of AS concentration (Subramanyam et al., 2013; Singh et al., 2017). The study showed optimized transformation protocol was able to produce putative transgenic patchouli plants harboring the *ACC deaminse* gene with durability 52.0 ± 0.8% (**Figure 3I**) after hardening and acclimatization. The putatively transformed CIM-Samarth lines were screened at first by PCR for the existence of transgene and selection marker (**Figures 7A, B**). Similar studies have also been reported in *Ocimum gratissimum*, *Tagetes erecta*, and *Pelargonium graveolens* (Gupta and Rahman, 2015; Khan et al., 2015; Singh et al., 2017). In this research, (**Figure 7C**) signifies that PT4 showed a high relative expression of *ACC deaminase* transgene among other best-propagating transgenic lines. The variation in transgene expression among transgenic lines could be due to the constitutive CaMV 35S promoter activity influenced by several regulatory systems (Benfey and Chua, 1990).

Sustainable results were significant in building a homology model of our target gene and checking *in silico* docking affinity of ligand ACC with receptor *ACC deaminase.* We found that the Ramachandran plot of the model (**Figure 8**) showed 94.6% (539) of amino acid residues fell in the most favorable region. Similar data that validate the build model have also been mentioned by Pramanik et al. (2017). The best conformation of our docking result revealed four H-bonds between ACC and surrounding amino acids Cys-196, Val-198, Thr-199, and Gly-200 of the receptor (**Figures 4C-F**) These studies were equivalent to other literature (Vijesh et al., 2013; Jasim et al., 2015). Additionally, as per Singh and Kashyap et al. (2012), few other amino acids that are conserved in other ACC deaminase sequences interact with ACC.

In this investigation, we have created a severe drought stress experiment where our best transgenic patchouli line PT4 confirmed by different molecular analyses was able to survive without compromising other physiological parameters such as RWC, chlorophyll content, and ROS production (**Figures 9**, **5**). This result correlates with previous reports indicating that under-stress plants develop mechanisms to maintain water retention and transpiration rate ratio (Merah, 2001; Soltys-Kalina et al., 2016). Our study mentioned ~75% of RWC in the transgenic line as opposed to to the control plant’s ~58% in severe stress conditions. However, an earlier study of *Pelargonium graveolens* RWC of transgenic was 88% in severe stress (Singh et al., 2021). Additionally it has been analyzed that the result depends on various properties of plants such as plant age, species, growth condition, and pot size. Elevated RWC in transgenic lines after severe watered stress over control plants becomes significant in drought conditions. Chlorophyll, a photosynthetic pigment, has a significant role in photosynthesis therefore variation in chlorophyll content may damage the plant photosystem. In our result, chlorophyll content decreased to ~2.3 fold in WT compared to transgenic plants that showed a ~1.6 fold decrease. This denotes that suitable transgenic lines can deal with drought stress without hampering the photosynthetic system. Similar strategies by Afridi et al. (2019); Han et al. (2017), and Bahieldin et al. (2005) have also suggested the same relationship between chlorophyll content and the rate of photosynthesis. Literature surveys suggest, during various biochemical reactions, the ROS produced in different organelles has an important role in growth, development, and function as a defense system in abiotic stress. Moreover, if excess ROS cannot detoxify by antioxidants, it leads to oxidative stress (Apel and Hirt, 2004; Tripathy and Oelmüller, 2012), which causes versatile cellular damage and may even cause the death of the plant. In our finding, DAB and NBT histochemical analysis of transgenic *P. cablin* leaves were showing less magnified brown and blue spots respectively compared to WT under severe stress conditions (**Figure 5**) That indicated less ROS (H_2_O_2_, O_2_^-^) production in transgenic plants expressing *ACC deaminase* gene. Similar research has also been reported on *Petunia hybrida* and *Pelargonium graveolens* (Singh et al., 2021; Naing et al., 2022).

## 5 Conclusion

Our research is novel in establishing an efficient direct regeneration and genetic transformation protocol using tTCL in only 15-20 days, which effectively enhances *Agrobacterium*-mediated genetic transformation frequency in *Pogostemon cablin*. tTCL sections regenerated faster than leaf and petiole explants, which improves micropropagation by reducing transgenic development time. The comprehensive study is the first report of *ACC deaminase* integration into the genome for developing transgenic patchouli to deal with drought stress, one of the significant problems in the propagation of aromatic plant ‘patchouli’. There is no report on the physiological parameters RWC, H_2_O_2_, and chlorophyll content of transgenic patchouli to support improved drought tolerance than wild-type plants. In our work, an *in-silico* study revealed better ligand interaction with the active site amino acid of the receptor. The regeneration and transformation protocol provides a platform for reverse genetics and helps in the modulation of metabolic pathways to enhance secondary metabolites and PO yield. Furthermore, the heterologous expression of *ACC deaminase* will support the management of PO global demand and develop a superior drought-tolerant variety of *P. cablin* plants.

## Data availability statement

The original contributions presented in the study are included in the article/supplementary material. Further inquiries can be directed to the corresponding author.

## Author contributions

ZW: Conceptualisation, Methodology, Visualization, Investigation, Data curation, Formal analysis, Writing-original draft, Writing- review & editing, Insilico data analysis, Validation. KK: Writing-review & editing, Formal analysis, Methodology, Insilico data analysis, conceptualisation, Validation. PS: review & editing, SEM analysis, provide ACC deaminase construct. LR: Conceptualisation, Investigation, Methodology, Supervision, Writing and editing. All authors contributed to the article and approved the submitted version
